# Auxin decreases chromatin accessibility through the TIR1/AFBs auxin signaling pathway in proliferative cells

**DOI:** 10.1038/s41598-018-25963-y

**Published:** 2018-05-17

**Authors:** Junko Hasegawa, Takuya Sakamoto, Satoru Fujimoto, Tomoe Yamashita, Takamasa Suzuki, Sachihiro Matsunaga

**Affiliations:** 1 Department of Applied Biological Science, Faculty of Science and Technology, Tokyo University of Science, 2641 Yamazaki, Noda, Chiba, 278-8510 Japan; 2 College of Bioscience and Biotechnology, Chubu University, 1200 Matsumoto-cho, Kasugai, Aichi 487-8501 Japan

## Abstract

Chromatin accessibility is closely associated with chromatin functions such as gene expression, DNA replication, and maintenance of DNA integrity. However, the relationship between chromatin accessibility and plant hormone signaling has remained elusive. Here, based on the correlation between chromatin accessibility and DNA damage, we used the sensitivity to DNA double strand breaks (DSBs) as an indicator of chromatin accessibility and demonstrated that auxin regulates chromatin accessibility through the TIR1/AFBs signaling pathway in proliferative cells. Treatment of proliferating plant cells with an inhibitor of the TIR1/AFBs auxin signaling pathway, PEO-IAA, caused chromatin loosening, indicating that auxin signaling functions to decrease chromatin accessibility. In addition, a transcriptome analysis revealed that several *histone H4* genes and a histone chaperone gene, *FAS1*, are positively regulated through the TIR1/AFBs signaling pathway, suggesting that auxin plays a role in promoting nucleosome assembly. Analysis of the *fas1* mutant of *Arabidopsis thaliana* confirmed that FAS1 is required for the auxin-dependent decrease in chromatin accessibility. These results suggest that the positive regulation of chromatin-related genes mediated by the TIR1/AFBs auxin signaling pathway enhances nucleosome assembly, resulting in decreased chromatin accessibility in proliferative cells.

## Introduction

Chromatin functions depend on the accessibility of chromatin-interacting factors and chromatin affecters. For example, the activation of transcription and DNA repair require the facilitated access of transcription factors and DNA-repairing factors to chromatin^1^. In contrast, access of DNA-damaging factors including reactive oxygen species (ROS) and chemicals must be restricted to prevent DNA damage^2,3^. The accessibility of these factors to chromatin is highly associated with chromatin structure, which is tightly regulated by chromatin-related proteins including histone variants, histone chaperones, histone-modifying enzymes, and chromatin remodeling factors^4–7^. Therefore, the appropriate adjustment of chromatin accessibility by chromatin-related proteins is fundamental for healthy plant growth^8,9^ and survival under stress conditions^10,11^.

The possibility of a relationship between plant hormone signaling and chromatin accessibility has been suggested by analyses of genes involved in plant hormone signaling, transport, and biosynthesis^12–14^. For example, in *Arabidopsis thaliana*, the recruitment of SWI2/SNF2 chromatin remodeling ATPases to chromatin by the MONOPTEROS transcription factor increases the accessibility of additional transcription factors to DNA to initiate the transcription of genes involved in flower primordium development under high-auxin conditions^15^. The SWI2/SNF2 chromatin remodeling ATPase, BRAHMA, was shown to directly regulate *PIN-FORMED* genes encoding auxin transporters to maintain the root stem cell niche^16^. The regulation of *YUCCA* genes involved in auxin biosynthesis is controlled by LIKE HETEROCHROMATIN PROTEIN 1, a homolog of metazone HETEROCHROMATIN PROTEIN 1^17^. Plant hormones have been shown to affect the expression of histone genes in several plant species. A gibberellic acid treatment was shown to enhance the expression of the histone *H3* gene in rice and the histone *H1* and *H2B* genes in tomato^18,19^^.^ Other studies showed that abscisic acid treatments increased the expression of histone *H1* in tomato and tobacco^20,21^ and that a methyl jasmonate treatment repressed the expression of histone *H2B* and *H4* genes in hot pepper^22^. Furthermore, the expression of the histone *H4* gene decreased under auxin-starvation conditions in cultured tobacco BY-2 cells^23^, and increased in response to an auxin treatment in hot pepper^22^. However, the biological significance of plant hormones in the regulation of chromatin accessibility remains unclear.

In this study, we focused on auxin and investigated its function in chromatin accessibility, because it is relatively easy to create auxin-starved conditions for tobacco BY-2 cells that cannot biosynthesize auxin^24^ and to disturb auxin signaling using chemicals^25^. The use of an almost homogenous population of cultured plant cells in these analyses allowed us to discuss the cellular responses in terms of regulation of chromatin accessibility by auxin. Our results demonstrate that auxin reduces chromatin accessibility through the TIR1/AFB pathway in proliferative cells.

## Results

### Auxin starvation affects chromatin accessibility in tobacco BY-2 cells

**Figure 1:**
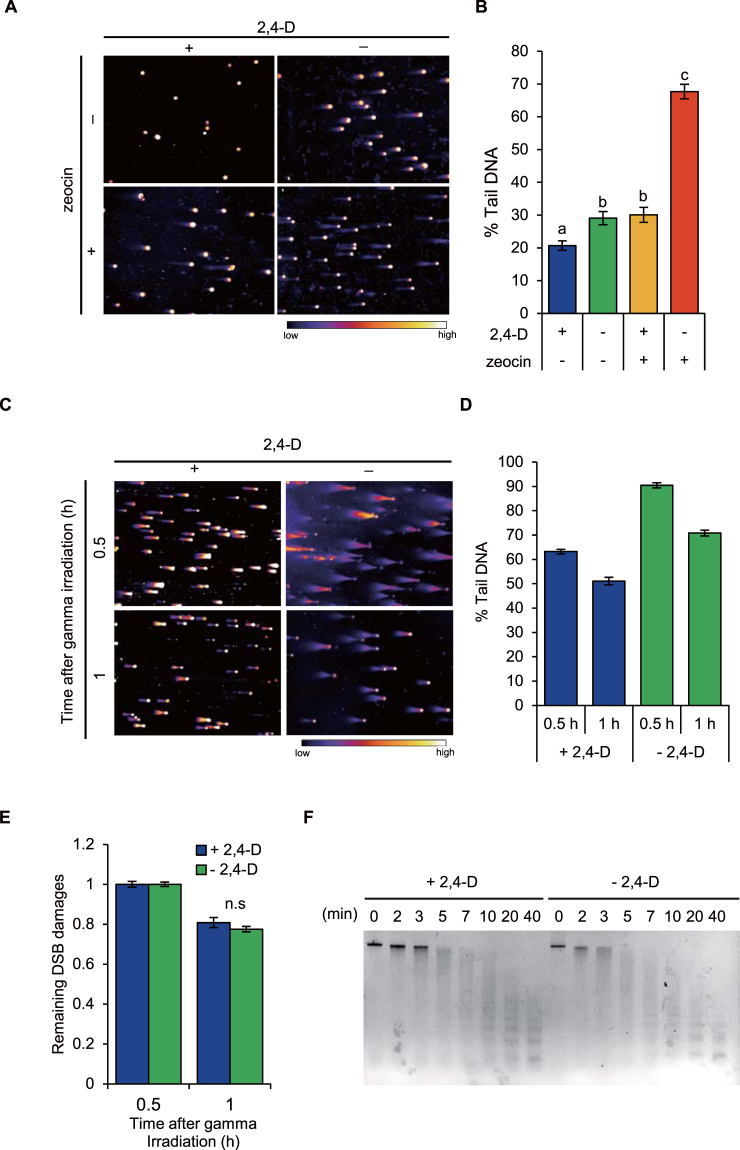
Effect of auxin deficiency on DNA double strand breaks (DSBs) induction in tobacco BY-2 cells. (**A**) Representative images of nuclei subjected to neutral comet assay after 2 days of auxin starvation and 50 μM zeocin treatment. The nuclei were stained with SYBR Green I. A colormap indicates the relative intensity of the fluorescence. (**B**) Levels of DSBs represented by % Tail DNA. Levels of DSBs were quantified using images of nuclei shown in (**A**). Values are means ± SE (50 < *n*). Different letters on bars indicate significant differences (*p* < 0.01; Steel–Dwass multiple comparison test). (**C**) Representative images of nuclei subjected to neutral comet assay under auxin-starved and γ-irradiated conditions. After 2 days of auxin deficiency, cells were irradiated with γ-rays (100 Gy) using a ^137^Cs source (0.83 Gy/min). The nuclei were stained with SYBR Green I. A colormap indicates the relative intensity of the fluorescence. (**D**) Levels of DSBs represented by % Tail DNA. Levels of DSBs were quantified using images of nuclei shown in (**C**). Values are means ± SE (100 < *n*). (**E**) Rate of DSBs repair after transient DSB induction by γ-irradiation. Rates are expressed as relative % Tail DNA remaining over time. Values of % TAIL DNA at 0.5 h after treatment are defined as 1. Values are means ± SE (100 < *n*). There was no significant difference between 2, 4-D-sufficient or -deficient conditions (Two-tailed student’s *t*-test). (**F**) Evaluation of chromatin accessibility under auxin-deficient conditions by micrococcal nuclease (MNase) assay. After 2 days of auxin deficiency, extracted chromatin was digested with 0.5 U MNase for indicated times. Equal amounts of DNA (1.6 μg) were electrophoresed.

An increase in chromatin accessibility is known to increase susceptibility to DNA-damaging factors^3^. Based on this relationship, we considered the change in the level of damaged DNA to represent the change in chromatin accessibility in this study. To evaluate whether auxin signaling affects chromatin accessibility, we first investigated the effects of auxin starvation on the accumulation of DNA double strand breaks (DSBs) under DSB-inducing conditions. Here, we used the plant cell line tobacco BY-2, which is auxin-auxotrophic and lacks an auxin biosynthesis system^24^. This allowed us to create an experimental system with depleted endogenous auxin by using growth medium without auxin. The levels of DSBs in cells cultured with a DSB-inducing chemical, zeocin^26^, under auxin-starved conditions were quantified by a comet assay^26^. The comet assay is known as single cell gel electrophoresis in which the damaged DNA migrates out of the nucleus forming a tail. The extent of the tail correlates with the level of DNA damage. Zeocin treatment caused additional accumulation of DSBs in BY-2 cells both in auxin-replete and auxin-starvation conditions (Fig. 1A,B). The increase in DSB levels in response to zeocin treatment was ~2.5 fold higher in auxin-starved conditions than in auxin-replete conditions (Fig. 1B). Interestingly, auxin starvation alone caused a ~1.5-fold increase in DSBs accumulation, comparable to the increase after zeocin treatment (Fig. 1B). These results indicated that auxin signaling affects the accumulation of DSBs irrespective of exogenous DSB induction in tobacco BY-2 cells.

Two processes can result in the increased accumulation of DSBs: increased accessibility of DSBs-inducing factors to chromatin; and reduced activity of the repair of damaged DNA. To evaluate the involvement of the latter process in the increased DSBs accumulation by auxin starvation, we compared the velocity of DNA repair of DSBs transiently induced by γ-irradiation under auxin-replete and auxin-starved conditions. The tobacco BY-2 cells were irradiated at 100 Gy, and then the recovery of DNA damage was monitored (Fig. 1C,D). Like in the zeocin treatment, auxin starvation caused a ~1.5-fold increase in additional DSB accumulation at both 30 min and 1 h after irradiation (Fig. 1C,D). When we compared the proportion of DSBs remaining at 1 h after irradiation relative to the %Tail DNA at 0.5 h after irradiation, there was no significant difference between auxin-replete and auxin-starved conditions, showing that DNA repair occurred at the same velocity regardless of auxin (Fig. 1E). This result suggested that auxin starvation increases the susceptibility of chromatin to DSBs-inducing factors and does not reduce the DNA repair activity.

Finally, we directly investigated the effect of auxin starvation on chromatin accessibility using a micrococcal nuclease (MNase) assay. MNase preferentially cleaves accessible linker DNA between two neighboring nucleosomes. Therefore, the sensitivity of chromatin DNA to cleavage by MNase is a direct indicator of the extent of chromatin accessibility. In auxin-starved conditions, the chromatin was mostly digested after 10 min of a MNase treatment, while some undigested chromatin was still detectable even after a 40 min MNase treatment in auxin-replete conditions (Fig. 1F), confirming that auxin starvation causes increased chromatin accessibility in tobacco BY-2 cells. Taken together, our results obtained from BY-2 cells suggested that auxin signaling functions to reduce chromatin accessibility, or in other words, auxin promotes chromatin condensation.

### Involvement of TIR1/AFBs pathway in regulation of chromatin accessibility in MM2d cells

**Figure 2:**
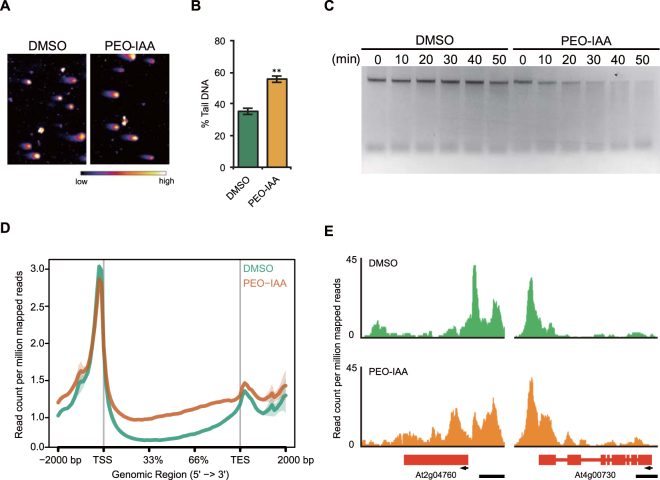
Effect of inhibition of TIR1/AFBs pathway on chromatin accessibility in MM2d cultured cells. (**A**) Representative images of nuclei subjected to neutral comet assay after 10 μM PEO-IAA treatment of cells for 2 days. The nuclei were stained with SYBR Green I. Color bar indicates the relative intensity of the fluorescence. (**B**) Levels of DSBs represented by % Tail DNA. Levels of DSBs were quantified using images of nuclei shown in (**A**). Values are means ± SE (60 < *n*, **p < 0.01; Two-tailed student’s t-test). (**C**) Evaluation of chromatin accessibility after 10 μM PEO-IAA treatment by micrococcal nuclease (MNase) assay. After 2 days PEO-IAA treatment, extracted chromatin was digested with 0.25 U MNase for indicated times. Equal amounts of DNA were electrophoresed. (**D**) Evaluation of distribution of accessible chromatin after 10 μM PEO-IAA treatment by ATAC-seq analysis. After 2 days PEO-IAA treatment, extracted chromatin was subjected to ATAC-seq analysis. The distribution of open accessible chromatins from 2 kb upstream to 2 kb downstream of gene body region (from the first exon to the last exon) is shown. This experiment was performed twice and similar result was obtained. TSS; transcriptional start sites. TTS; transcriptional termination sites. (**E**) Representative genes with higher enrichment of ATAC-seq signals in gene body region in PEO-IAA treated nuclei. Bars; 1 kb.

Next, we analyzed the involvement of auxin signaling in the regulation of chromatin accessibility. Auxin signaling is mainly classified into two pathways; TIR1/AFBs-mediated auxin signaling^27,28^ and the TIR1/AFBs-independent pathway^29^. So far, it is unclear whether changes in chromatin accessibility are regulated through TIR1/AFBs-mediated auxin signaling and/or another auxin signaling pathway. In a previous study, the F25 useful auxin antagonist PEO-IAA was shown to bind to TIR1/AFBs auxin receptors in *A*. *thaliana* and inhibit TIR1/AFBs-mediated auxin signaling^25^. Here, we verified the involvement of TIR1/AFBs-mediated auxin signaling in the regulation of chromatin accessibility by using PEO-IAA and a cultured cell line of *A*. *thaliana*, MM2d. The results of the comet assay revealed that PEO-IAA treatment caused a ~1.6-fold increase in DSBs accumulation (Fig. 2A,B). In addition, PEO-IAA treatment increased the chromatin sensitivity to MNase as shown by the faster digestion of PEO-IAA-treated chromatin (Fig. 2C). The results of ATAC-seq, which reveals accessible chromatin regions by using the action of Tn5 transposase that causes DNA cleavage and simultaneous insertion of sequencing adapters into open chromatin regions^30^, confirmed that PEO-IAA treatment indeed increased chromatin accessibility, especially in the gene body region (Fig. 2D,E). Although we cannot exclude the possible involvement of another auxin signaling pathway, these results suggested that auxin acts in the regulation of chromatin accessibility through the TIR1/AFBs-mediated auxin signaling pathway.

### Involvement of TIR1/AFBs pathway in expression of chromatin accessibility-related genes in MM2d cells

**Figure 3:**
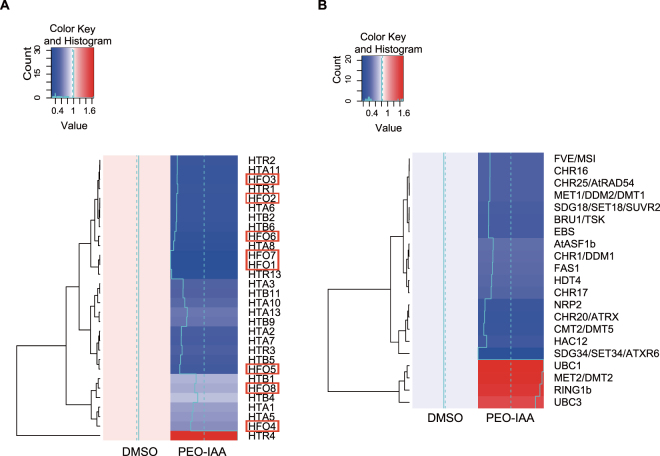
Effect of inhibition of TIR1/AFBs pathway on expression of genes related to chromatin accessibility in MM2d cultured cells. (**A**,**B**) Effect of 10 μM PEO-IAA treatment on gene expression in MM2d cells. After 6 h PEO-IAA treatment, extracted RNA was subjected to RNA-seq analysis. Histone genes (**A**) and chromatin-related genes (**B**) showing significant changes in expression (*p* < 0.01) are shown (*n* = 3). Genes enclosed in a red flame are the paralogues of *histone H4*.

Auxin is known to regulate gene expression through TIR1/AFBs-mediated signaling^4,25,28^. Therefore, we presumed that the regulation of genes related to chromatin accessibility would be downstream of the TIR1/AFBs pathway. To identify such genes, we analyzed gene expression in MM2d cells treated with PEO-IAA by RNA-seq. We found that PEO-IAA treatment significantly altered the expression of 3833 genes (*p* < 0.01): 1222 of them were down-regulated (PEO-IAA/DMSO ≤0.67-fold) and 1434 were up-regulated (Supplementary Fig. S1, Dataset S1). Interestingly, the GO analysis showed a significant enrichment of down-regulated genes especially in the categories ‘chromosome organization’ and ‘chromatin organization’ in addition to ‘one-carbon metabolic process’, ‘methylation’ and ‘macromolecule methylation’ (Dataset S2). Next, we focused on histone genes and genes related to chromatin remodeling, which are encoded at 50 and 236 loci in *A*. *thaliana* genome, respectively^31–33^. The expression levels of 23 histone genes and 17 chromatin remodeling-related genes were down-regulated by PEO-IAA treatment (PEO-IAA/DMSO ≤0.67), and those of 1 histone gene and 4 chromatin remodeling-related genes were up-regulated by the treatment (1.5≤ PEO-IAA/DMSO) (Fig. 3A,B). All 8 genes encoding core histone H4 were down-regulated, and 6 of them showed expression levels less than half of their original expression levels in response to PEO-IAA treatment (Fig. 3A). Consistent with this, the PEO-IAA treatment also decreased the level of histone H4 protein (Supplementary Fig. S2). The PEO-IAA treatment also resulted in decreased expression levels of some known histone chaperone genes related to nucleosome organization, including *CHR20* (*ATRX*), *NRP2*, *FAS1*, *AtASF1a*, and *SUVR2*^34,35^ (Fig. 3B).

### Involvement of TIR1/AFBs pathway in regulation of chromatin accessibility in the root apical meristem of *A*. *thaliana*

**Figure 4:**
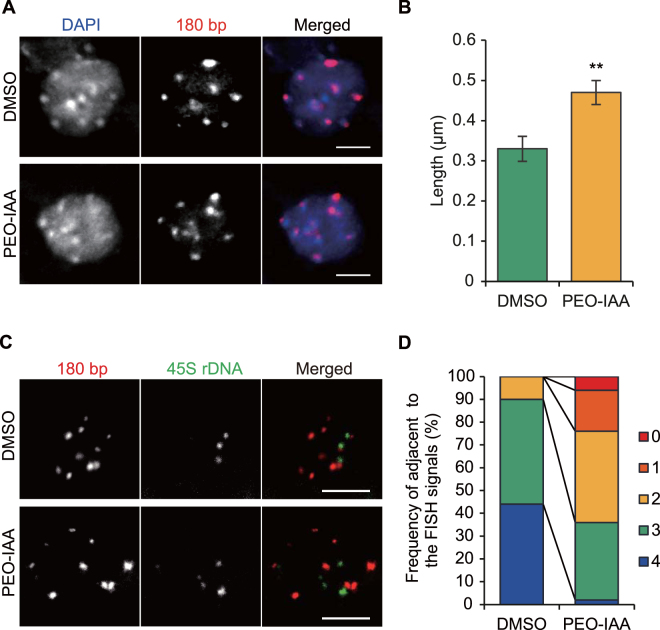
Effect of inhibition of TIR1/AFBs pathway on chromatin accessibility in root apical meristem of *A. thaliana*. (**A**) Representative images of 180-bp repeat visualized with TALE_180-tdTomato (red) and chromocenter stained with DAPI (blue) in nuclei of root meristem cells from seedlings treated with 10 μM PEO-IAA for 2 days. Scale bar; 5 µm. (**B**) Effect of 10 μM PEO-IAA treatment on distance between 180bp-tdTomato signals and chromocenter signals shown in (**A**) (*n* = 103) (**p < 0.01; Two-tailed student’s *t*-test). (**C**) Representative images of 180-bp repeat (magenta) and 45S rDNA (green) loci visualized by FISH in nuclei of root meristem cells from seedlings treated with 10 μM PEO-IAA for 2 days. Scale bar; 5 µm. (**D**) Effect of 10 μM PEO-IAA treatment on number of 45S rDNA signals overlapping with 180-bp repeat locus (*n* = 50). Normally, there are four 45S rDNA signals in nucleus. Frequency of the number of 45S rDNA signals overlapping with 180-bp repeat locus is shown. The overlap was analyzed using images shown in (**C**).

Our data showed that chromatin accessibility was increased by inhibition of the TIR1/AFBs pathway in cultured plant cells that maintained meristematic activity. Next, we examined whether the inhibition of the TIR1/AFBs pathway increases chromatin accessibility in the root apical meristem (RAM) of *A*. *thaliana* seedlings. Because chromatin loosening results in increased chromatin accessibility, we assessed the extent of chromatin loosening in the RAM by observing the positional relationship between two distinct major DNA loci that usually exist in close proximity; the chromocenter and the pericentromeric region. We visualized the chromocenter by 4′,6-diamidino-2-phenylindole (DAPI) staining. The pericentromeric region was visualized by detecting 180-bp repeats, which are located in this region, using TALE_180-tdTomato^36^ and fluorescence *in situ* hybridization (FISH), which was also used to detect 45S rDNA loci, which overlap with the pericentromeric region^37^. The PEO-IAA treatment increased the distance between the 180-bp repeats and the closest chromocenter (Fig. 4A,B). Similarly, the number of 45S rDNA signals separated from the 180-bp repeat signals was increased after 10 μM PEO-IAA treatment (Fig. 4C,D). These results indicated that chromatin accessibility is increased by the inhibition of the TIR1/AFBs pathway in root meristematic cells of *A*. *thaliana* seedlings.

### Involvement of FAS1 in TIR1/AFBs pathway-dependent regulation of chromatin accessibility in the RAM of *A*. *thaliana*

Next, we focused on genes downstream of the TIR1/AFBs pathway that may be involved in the regulation of chromatin accessibility in the RAM of *A*. *thaliana*. Our transcriptome analysis of MM2d cells revealed that *FAS1* was among the genes down-regulated by the PEO-IAA treatment (Fig. 3B). FAS1 is a component of the Chromatin Assembly Factor 1 complex, and is a H3/H4 histone chaperone responsible for replication-dependent nucleosome assembly^38^. The *fas1* mutant has been shown to accumulate DSBs resulting from both endogenous and exiguous genotoxic stresses^39–41^, indicating the significance of FAS1 in suppressing chromatin loosening^4,38^. Therefore, we speculated that FAS1 may be a key factor determining chromatin accessibility under the regulation of the TIR1/AFBs pathway in the RAM.

**Figure 5:**
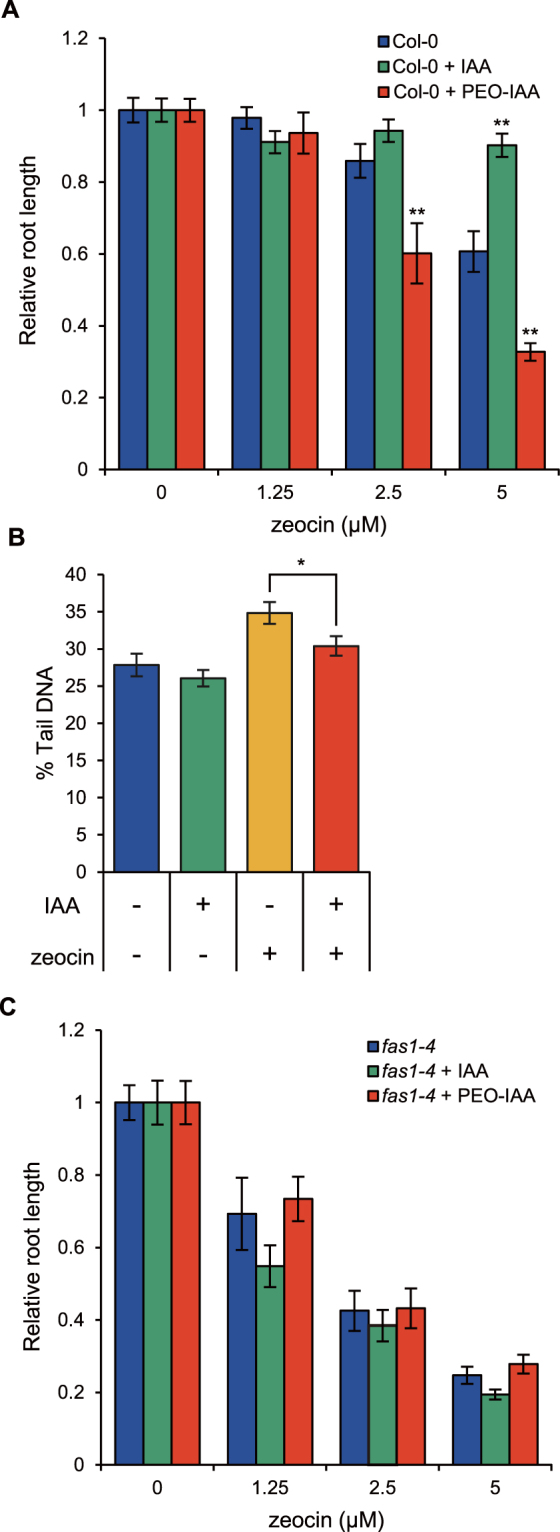
Analysis of FAS1 function in TIR1/AFBs-dependent regulation of chromatin accessibility. (**A**) Effects of 5 nM IAA and 5 μM PEO-IAA on inhibition of root elongation caused by zeocin treatment in wild-type Col-0. Five-day-old seedlings were treated with drugs for 6 days. Root length is expressed as ratio relative to that of untreated seedlings (defined as 1). Values are means ±SE (17 < *n*, **p < 0.01; Dunnett’s test). (**B**) Levels of DSBs represented by % Tail DNA in RAM of *A*. *thaliana* treated with 2.5 μM zeocin and 5 nM IAA. Five-day-old seedlings were treated with drugs for 2 days. Levels of DSB were quantified using images of assayed nuclei. Values are means ± SE (100 < *n*) (*p < 0.05; Two-tailed student’s *t*-test). (**C**) Effects of 5 nM IAA and 5 μM PEO-IAA on inhibition of root elongation caused by zeocin treatment in *fas1-4*. Five-day-old seedlings were treated with drugs for 6 days. Root length is expressed as ratio relative to that of untreated seedlings (defined as 1). Values are means ± SE (16 < *n*). The absolute value of root elongation is shown in Supplementary Fig. S3.

To test this possibility, we indirectly assessed chromatin accessibility in roots. The accumulation of DSBs in the RAM inhibits root elongation in a dose-dependent manner^26^; therefore, we assumed that the sensitivity of root elongation to zeocin reflects the extent of chromatin accessibility in the RAM. We found that the sensitivity of root elongations to 2.5 and 5 μM zeocin was increased by the PEO-IAA treatment (Fig. 5A, Supplementary Fig. S3), confirming that the increase in chromatin accessibility strengthens the sensitivity to DSBs. In contrast, treatment with an auxin, indole acetic acid (IAA) suppressed the inhibitory effect of 5 μM zeocin on root elongation. The accumulation level of DSBs in the root tips was lower in the presence of IAA than in IAA-free conditions (Fig. 5B, Supplementary Fig. S3), indicating that the IAA treatment reduced chromatin accessibility in the RAM. These results suggested that chromatin accessibility in the RAM varies in response to the activity of the TIR1/AFBs pathway, which correlates with the extent of root elongation under DSBs-inducing conditions.

Finally, we investigated the involvement of FAS1 in the TIR1/AFBs-dependent regulation of chromatin accessibility in the RAM by using the *fas1* mutant, *fas1-4*. *fas1-4* was much more sensitive to DSBs induction by zeocin treatment than was the wild type Col-0 treated with PEO-IAA (Fig. 5A,C). In contrast to Col-0, *fas1-4* did not show increased sensitivity to zeocin after PEO-IAA treatment. In addition, the IAA treatment did not rescue the root elongation of *fas1-4* in any of the zeocin treatments (Fig. 5C, Supplementary Fig. S3). These results indicated that FAS1 functions downstream of the TIR1/AFBs pathway in the RAM of *A*. *thaliana*.

## Discussion

On the basis of our results, we propose a novel relationship between the auxin signaling pathway and chromatin-related proteins. Several studies have shown that chromatin-related proteins can regulate auxin responses^4,15–17,42–44^, but none has shown that auxin regulates chromatin-related proteins. Our results suggest that auxin, through the TIR1/AFBs auxin signaling pathway, upregulates several genes encoding chromatin-related proteins that maintain genomic stability by reducing chromatin accessibility in proliferating plant cells.

It is still unknown how the chromatin-related genes affected by the PEO-IAA treatment (Fig. 3A,B) are regulated through the TIR1/AFBs pathway. TIR1/AFBs is known to activate transcription factors, which include auxin response factors (ARFs) that recognize the auxin-response element (AuxRE) in the promoter regions of auxin-response genes and activate or suppress their expression^25^. Interestingly, we found that several genes responsive to PEO-IAA had AuxREs in the upstream regions of their loci (Supplementary Table S2), implying their direct regulation by ARFs. To date, however, no ARFs have been reported to interact with those gene loci. To explore the details of the regulatory mechanism, further analyses such as reporter–effector transient assays against the promoter regions of those genes are required.

Inhibition of auxin responses increased chromatin accessibility in both tobacco BY-2 and MM2d cells (Figs 1F and 2C,D). There are two processes that could result in increased accessibility of linker DNA between two neighboring nucleosomes: a reduction in nucleosome occupancy; and reduced interaction between DNA and histones by epigenetic regulation such as histone acetylation. Auxin starvation was shown to repress the expression of *histone H4*, encoding a core histone for nucleosome conformation, in tobacco BY-2 cells^23^. Consistent with this, we found that the PEO-IAA treatment led to reduced levels of the transcriptional and translational products of *histone H4* in MM2d cells (Fig. 3A, Supplementary Fig. S5). In addition, FAS1 was identified as an essential factor for increased chromatin accessibility in response to activity of the TIR1/AFBs pathway in the RAM of *A*. *thaliana* (Figs 3B and 5). Considering that FAS1 is required to assemble nucleosomes for replicated DNA^45^, these data imply that the inhibition of auxin responses principally causes a reduction in nucleosome occupancy. We note that the inhibition of auxin signaling also affected the histone acetylation status, as both histone acetylases and deacetylases were down-regulated by the PEO-IAA treatment (Fig. 3A). The change in histone acetylation status would further destabilize chromatin. Moreover, the fact that genes having GO terms involved in DNA methylation were down-regulated by the PEO-IAA treatment (Dataset 2) led us to speculate that auxin signaling has effects on organization of transcriptionally repressive chromatin such as heterochromatin through changes in the DNA methylation status.

Our results demonstrated auxin-dependent changes in chromatin accessibility in the RAM (Figs 4, 5). In the RAM, the auxin maximum is around quiescent center (QC) and initial stem cells, in which cells divide very infrequently, suggesting that there is low chromatin accessibility in these cells. Similarly, a FRAP analysis of H2B-GFP confirmed reduced chromatin accessibly in the stem cell niche^46^. The less chromatin accessibility would contribute to keep the activity of cell division low in QC and initial stem cells. In contrast, meristematic cells except for QC and initial cells still have relatively high auxin level and show high cell division rate. Therefore, there might be a threshold of auxin level that coordinates with cell division. In other words, auxin-dependent modulation of chromatin accessibility might regulate the rate of cell division in proliferative cells. On the other hand, as the auxin level in *A*. *thaliana* root is known to decrease toward differentiation^47^, chromatin accessibility is anticipated to be high in differentiated cells. However, the acetylation level of histone H4, which is a well-known mark for chromatin loosening, is lower in differentiated cells compared to meristematic cells in *A*. *thaliana* root^46^, indicating less chromatin accessibility in differentiated cells. It is hence considered that auxin-dependent chromatin regulation mainly works in proliferative cells and there might be other mechanisms that affect chromatin accessibility in differentiated cells.

Environmental stresses such as aluminum, cadmium, and excess boron stress, have been shown to increase auxin levels in the RAM of *A*. *thaliana*^48–51^. In addition, those stresses are potentially genotoxic^26,52,53^. Our finding that auxin treatment confers tolerance to DNA damage in the RAM (Fig. 5) suggests that increased auxin accumulation could be a defensive response against genotoxicity to protect the genome by reducing chromatin accessibility and to arrest the cell cycle to prevent the inheritance of genomic mutations caused by DNA damage to daughter cells during cell proliferation.

In conclusion, our results have revealed new aspects of auxin function in regulating chromatin accessibility, which is closely associated to gene regulation and chromatin integrity. Considering that both transient and constitutive auxin accumulation often occur during development and in response to environmental stimuli in various plant tissues, auxin-dependent regulation of chromatin accessibility might play crucial roles in gene regulation and chromatin protection under stress conditions.

## Methods

### Materials and growth conditions

The *fas1-4* line (SAIL-662-D10; background, Col-0) was obtained from the Arabidopsis Biological Resource Center (ABRC). Plants harboring TALE_180-tdTomato were generated as described previously^36^. Seeds were germinated and vertically grown on medium [1/2 MS (Wako, Osaka, Japan), 1% (w/v) sucrose, 1% (w/v) gellan gum, pH 5.8 adjusted with KOH] in a plant growth chamber under a 16-h light/ 8-h dark cycle at 22 °C.

The tobacco BY-2 cell line was maintained as described previously^54^. The growth conditions and medium were the same for the MM2d cell line and the tobacco BY-2 cell line. As for MM2d cells, they were cultured in the presence of 2, 4-D in all experiments. A proportion of both types of cultured cells was transferred to new medium once a week. Cells were cultured with rotary shaking at 130 rpm at 27 °C in the dark.

### Drug treatments to cultured cells

Drug treatments were applied to tobacco BY-2 cells as described previously^54^. Seven-day-old tobacco BY-2 cells were washed twice with five volumes of 2, 4-D-free LS modified medium^55^ and then washed cells (2 mL) were transferred to 95 mL medium containing 0 or 50 µM zeocin, and/or 0.2 mg/mL 2, 4-D. The cells were cultured for a further 2 days.

For MM2d cells, 2-day-old cells were treated with DMSO or 10 µM PEO-IAA^25^ and then cultured for a further 6 h or 2 days.

### Root elongation test

Seedlings grown for 5 days after vernalization were transferred to medium containing 5 nM IAA, 5 μM PEO-IAA, or indicated concentrations of zeocin. At 6 days after transfer, the length of the elongated primary root was measured using ImageJ software (http://rsb.info.nih.gov/ij/).

### Assessment of positional relationship between two distinct DNA loci

TALE_180-tdTomato-expressing plants grown on solid medium for 5 days after vernalization were transferred to liquid medium (1/2 MS, 1% sucrose pH 5.8 adjusted with KOH; containing DMSO or 10 µM PEO-IAA). After 2 days incubation in the liquid medium, seedlings were stained with DAPI and mounted with 50% TDE solution^56^. TALE_180-tdTomato-expressing plants were observed under an inverted confocal microscope (FV1200, Olympus, Tokyo, Japan) as previously described^36^. The distance between the TALE_180-tdTomato signal and the nearest chromocenter signal stained with DAPI was measured using ImageJ.

### Fluorescence *in situ* hybridization

The root tips (~1 cm) treated with 10 μM PEO-IAA for 2 days were fixed overnight in Fermer’s fixative at 25 °C, rinsed in 70% ethanol and distilled H_2_O, and then incubated for 1 h at 37 °C in 500 µl enzyme solution (2% cellulose Onozuka RS, 0.5% Pectolyase Y-23 in citrate buffer). Then, the solution was mixed with 300 µl distilled H_2_O. The material was sequentially filtered through 100-µm and 30-µm nylon mesh. The filtrate was centrifuged (8000 rpm, 1 min) and the pellet was washed with Carnoy’s fixative. After centrifuging again, the pellet was resuspended in Carnoy’s fixative. The nuclei suspension was dropped on a glass microscope slide and air-dried. The slide was stored at −20 °C until use.

Prepared nuclei were treated with 30 µl RNase A (Roche, Basel, Switzerland) in 2 × saline sodium citrate (SSC) buffer for 30 min at 37 °C. After washing the slides in 2 × SSC for 15 min, nuclei were re-fixed for 5 min in 4% paraformaldehyde in PBS. After washing the slides in dH_2_O for 5 min, nuclei were dehydrated in an ethanol gradient from 70% to 100% ethanol (5 min each). After incubation for 30 min at 37 °C in a moisture chamber, the slides were soaked in SF (70% formamide in 2 × SSC) for 1 min at 76 °C, in 2 × SSC for 5 min at −20 °C, and then in 100% ethanol for 5 min at 25 °C. Subsequently, the nuclei were incubated in a moisture chamber for 30 min at 37 °C. To denature the labeled DNA, the probe was placed on a heating block for 10 min at 80 °C. For hybridization, 15 µl DNA probe was applied to the slide and incubated overnight in a moisture chamber at 37 °C. After hybridization, the slides were rinsed twice in 2 × SSC, and then in TNB (0.1 M Tris, 0.15 M NaCl, pH 7.5) for 5 min each. Subsequently, the nuclei were incubated for 30 min at 37 °C in 50 µl blocking solution (digoxigenin (DIG)-labeled probes and biotin-labeled probes were diluted 200 times with 1% blocking regent in TNB), which was directly applied to the nuclei. After washing with wash buffer (0.05% Tween 20 in TNB) containing DAPI (0.1 µg/mL) for 10 min, the nuclei were washed once with PBS for 5 min as the final preparation step before hybridization.

The DNA probes for FISH were synthesized as follows: Probes recognizing 180-bp centromeric repeats (forward primer; GATCAAGTCATATTCGACTC, reverse primer; GTTGTCATGTGTATGATTGA) or 45S rDNA (forward primer; CAAGCAAGCCCATTCTCCTC, reverse primer; CAACTAGACCATGAAAATCC) were synthesized by nick translation using DIG Nick or Biotin Nick Translation Mix (Roche). The DIG-labeled probes were visualized using anti-DIG-rhodamine Fab fragments (Roche) and biotin-labeled probes were visualized using Alexa Fluor 488 conjugated streptavidin (Thermo Fisher Scientific, Waltham, MA, USA). The cells were observed under a light microscope (BX53m, Olympus) equipped with a CCD camera (DOC CAM U3-50S5M-C, Molecular Devices, Tokyo, Japan). Overlapping 180-bp signals and 45s rDNA signals were analyzed by ImageJ.

### Neutral comet assay

For tobacco BY-2 cells and MM2d cells, the comet assay was conducted using a CometAssay® Kit (Trevigen, Gaithersburg, MD, USA) according to the manufacturer’s protocol. For the γ-irradiation treatment, 2-day-old tobacco BY-2 cells were irradiated with ^137^Cs source (100 Gy) (0.83 Gy/min; Research Institute for Biomedical Sciences, Tokyo University of Science). For *A*. *thaliana*, nuclei were isolated from root tips (~1 cm) treated with 10 μM PEO-IAA for 2 days and then assayed as described previously^57^. Nuclei observation and comet analysis were performed as described previously^57^.

### Micrococcal nuclease (MNase) assay

After the 2-day treatment to deplete auxin, nuclei were isolated by a modified protocol^58^. Cells (3 mL) were ground to a fine powder in liquid nitrogen using a mortar and pestle. Then, 3 mL Hamilton buffer (10 mM Tris-HCl pH 7.5, 1.14 M sucrose, 5 mM MgCl_2_, 5 mM β-mercaptoethanol) was added, and the mixture was filtered through a double layer of miracloth and centrifuged for 5 min at 2500 *g*. The supernatant was discarded and the pellet was resuspended in Hamilton buffer containing 0.15% (v/v) Triton X-100. After incubation for 15 min, the sample was centrifuged for 5 min at 2500 *g*. The pellet was resuspended in MNase digestion buffer (50 mM Tris-HCl, 0.3 M sucrose, 5 mM MgCl_2_, 1.5 mM NaCl, 3 mM CaCl_2_, 5 mM β-mercaptoethanol). Digestion was performed by adding 0.5 and 0.25 U/mL MNase (Takara, Shiga, Japan) to the nuclei suspension of BY-2 and MM2d, respectively. The mixture was incubated at 37 °C and aliquots (50 μl) were removed from the reaction mixture at indicated times. A double volume of stop buffer (20 mM EDTA pH 8.0, 20 mM Tris-HCl pH 8.0, 2% SDS, 0.205 U/mL Proteinase K) was added to stop the reaction. The mixtures were incubated for 30 min at 37 °C. The digested chromatin DNA was electrophoresed on a 1.5% agarose gel and the DNA concentration was determined by measuring the fluorescence derived from ethidium bromide staining using ImageJ.

### RNA extraction and RNA-seq analysis

Two-day-old MM2d cells were treated with DMSO or PEO-IAA for 6 h, and then total RNA was extracted using an RNeasy Plant Mini kit (Qiagen, Valencia, CA, USA). Complementary DNA libraries were constructed using TruSeq ® RNA Sample Prep Kit v2 (Illumina, San Diego, CA, USA) and were sequenced by NextSeq 500 (Illumina) in the single-end mode. Three biological replicates were analyzed. The produced bcl files were converted to fastq files by bcl2fastq (Illumina). The reads were analyzed as previously described^59^ and were mapped to the Arabidopsis reference (TAIR10) by bowtie 1.2.1^60^ with the following parameters: ‘-all -best -strata’ (Table S1). The number of reads mapped to each reference was counted. The sequencing data are deposited at DDBJ (accession number DRA005751).

### ATAC-seq

Two-day-old MM2d cells were treated with DMSO or PEO-IAA for 2 days, and then the nuclei were isolated as described previously^61^. Chromatin fragmentation and tagmentation were performed according to the standard ATAC-seq protocol^30^. The DNA libraries were sequenced by NextSeq 500 (Illumina) in the paired-end mode. The reads were mapped to the Arabidopsis reference by bowtie 1.2.1^60^ with the following parameters: ‘-m 3 –v 2’. Duplicated reads were removed using the default parameters of picard 2.17.11 (http://broadinstitute.github.io/picard/) (Table S1). MACS2 2.1.0^62^ was used to identify the accessible regions and peaks with the following parameters: ‘-nomodel -nolambda -keep-dup all -call-summits -q 0.01’. Output bedGraph format files were used to visualize accessible regions. The distribution of accessible chromatin was analyzed using ngs.plot^63^. Two biological replicates were analyzed and similar results were obtained. The sequencing data are deposited at DDBJ (accession number DRA006829).

### Western blotting

Nuclei were isolated as described below. After the 2-day PEO-IAA treatment, cells were frozen in liquid nitrogen and ground into a fine powder with a mortar and pestle. Then, 4 mL nuclei isolation buffer (10 mM HEPES pH 7.6, 1 M sucrose, 5 mM KCl, 5 mM MgCl_2_, 5 mM EDTA pH 8.0, 1 mM Pefabloc SC, complete solution) was added, and the mixture was filtered through a layer of Miracloth and centrifuged at 3000 *g* for 10 min at 4 °C. The pellet was resuspended in 300 µL nuclei isolation buffer. Then, the suspension was added to 500 µL nuclei separation buffer (10 mM HEPES pH 7.6, 1 M sucrose, 5 mM KCl, 5 mM MgCl_2_, 5 mM EDTA, pH 8.0, 15% Percoll) and the mixture was centrifuged at 3000 *g* for 5 min at 4 °C. The pellet containing the nuclei fraction was resuspended in nuclei isolation buffer. The protein concentration was determined using a Bio-Rad Protein Assay kit (Bio-Rad Laboratories, Hercules, CA, USA). Equal amounts of proteins were subjected to SDS-PAGE and subsequently transferred to a PVDF membrane (Immobilon-P, Millipore, Billerica, CA, USA). To detect histone H4, we used a 1:1000 dilution of anti-histone H4 rabbit IgG (ab10158, Abcam, Cambridge, MA, USA). The chemiluminescence of protein blots was detected using the Western Lightning ® ECL pro system (PerkinElmer, Waltham, MA, USA) on a Fusion Pulse instrument (Vilber Lourmat, Marine, France). After detecting chemiluminescence, the membrane was stained with colloidal gold solution.

### Bioinformatics and statistical analysis

Statistical analyses were performed using R (v3.2.0) open software. Gene ontology (GO) analysis was performed using agriGO^64^ (http://bioinfo.cau.edu.cn/agriGO/).

## Electronic supplementary material

Supplementary Information

Supplementary Dataset 1

Supplementary Dataset 2

## References

[1] Schneider R, Grosschedl R (2007). Dynamics and interplay of nuclear architecture, genome organization, and gene expression. Genes Dev..

[2] Falk M, Lukásová E, Kozubek S (2008). Chromatin structure influences the sensitivity of DNA to gamma-radiation. Biochim. Biophys. Acta.

[3] Takata H (2013). Chromatin compaction protects genomic DNA from radiation damage. PLoS One.

[4] Kirik A, Pecinka A, Wendeler E, Reiss B (2006). The chromatin assembly factor subunit FASCIATA1 is involved in homologous recombination in plants. Plant Cell.

[5] Zhu Y (2006). Arabidopsis NRP1 and NRP2 encode histone chaperones and are required for maintaining postembryonic root growth. Plant Cell.

[6] Toiber D (2013). SIRT6 recruits SNF2H to DNA break sites, preventing genomic instability through chromatin remodeling. Mol. Cell.

[7] Jégu T (2014). The BAF60 subunit of the SWI/SNF chromatin-remodeling complex directly controls the formation of a gene loop at FLOWERING LOCUS C in Arabidopsis. Plant Cell.

[8] Thorstensen T, Grini PE, Aalen RB (2011). SET domain proteins in plant development. Biochim. Biophys. Acta.

[9] Gentry M, Hennig L (2013). Remodelling chromatin to shape development of plants. Exp. Cell Res..

[10] Kim JM, Sasaki T, Ueda M, Sako K, Seki M (2015). Chromatin changes in response to drought, salinity, heat, and cold stresses in plants. Front. Plant Sci..

[11] Probst. AV, Mittelsten Scheid O (2015). Stress-induced structural changes in plant chromatin. Curr. Opin. Plant Biol..

[12] Alatzas A (2013). Histones and Plant Hormones: New evidence for an interesting interplay. Bot. Rev..

[13] Yamamuro C, Zhu JK, Yang Z (2016). Epigenetic modifications and plant hormone action. Mol. Plant.

[14] Weijers D, Wagner D (2016). Transcriptional responses to the auxin hormone. Annu. Rev. Plant Biol..

[15] Wu MF (2015). Auxin-regulated chromatin switch directs acquisition of flower primordium founder fate. Elife.

[16] Yang S (2015). The Arabidopsis SWI2/SNF2 chromatin remodeling ATPase BRAHMA targets directly to PINs and is required for root stem cell niche maintenance. Plant Cell.

[17] Rizzardi K, Landberg K, Nilsson L, Ljung K, Sundås-Larsson A (2011). TFL2/LHP1 is involved in auxin biosynthesis through positive regulation of YUCCA genes. Plant J.

[18] van der Knaap. E, Kende H (1995). Identification of a gibberellin-induced gene in deepwater rice using differential display of mRNA. Plant Mol. Biol..

[19] van den Heuvel KJ, van Esch RJ, Barendse GW, Wullems GJ (1999). Isolation and molecular characterization of gibberellin-regulated H1 and H2B histone cDNAs in the leaf of the gibberellin-deficient tomato. Plant Mol. Biol.

[20] Kahn TL, Fender SE, Bray EA, O'Connell MA (1993). Characterization of expression of drought- and ABAregulated tomato genes in the drought resistant species Lycopersicon pennellii. Plant Physiol..

[21] Wei T, O'Connell MA (1996). Structure and characterization of a putative drought-inducible H1 histone gene. Plant Mol. Biol..

[22] Kim SA, Kwak HJ, Park MC, Kim SR (1998). Induction of reproductive organ-preferential histone genes by wounding or methyl jasmonate. Mol. Cells.

[23] Miyazawa Y (2003). Activation of cell proliferation by brassinolide application in tobacco BY-2 cells: effects of brassinolide on cell multiplication, cell-cycle-related gene expression, and organellar DNA contents. J. Exp. Bot..

[24] Mano Y, Nemoto K (2012). The pathway of auxin biosynthesis in plants. J. Exp. Bot..

[25] Hayashi K (2012). Rational Design of an Auxin Antagonist of the SCF Auxin Receptor Complex. ACS Chem. Biol..

[26] Sakamoto T (2011). Condensin II alleviates DNA damage and is essential for tolerance of boron overload stress in Arabidopsis. Plant Cell.

[27] Gray WM, Kepinski S, Rouse D, Leyser O, Estelle M (2001). Auxin regulates SCF (TIR1)-dependent degradation of AUX/IAA proteins. Nature.

[28] Salehin M, Bagchi R, Estelle M (2015). SCF TIR1/AFB-based auxin perception: mechanism and role in plant growth and development. Plant Cell.

[29] Takahashi K, Hayashi K, Kinoshita T (2012). Auxin activates the plasma membrane H+-ATPase by phosphorylation during hypocotyl elongation in Arabidopsis. Plant Physiol..

[30] Buenrostro JD, Wu B, Chang HY, Greenleaf WJ (2015). ATAC-seq: A method for assaying chromatin accessibility genome-wide. Curr. Protoc. Mol. Biol.

[31] Okada T, Endo M, Singh MB, Bhalla PL (2005). Analysis of the histone H3 gene family in Arabidopsis and identification of the male-gamete-specific variant AtMGH3. Plant J.

[32] Pischke MS, Huttlin EL, Hegeman AD, Sussman MR (2006). A transcriptome-based characterization of habituation in plant tissue culture. Plant Physiol..

[33] Talbert PB (2012). A unified phylogeny-based nomenclature for histone variants. Epigenetics Chromatin.

[34] Han YF (2014). SUVR2 is involved in transcriptional gene silencing by associating with SNF2-related chromatin-remodeling proteins in Arabidopsis. Cell Res..

[35] Tripathi AK, Singh K, Pareek A, Singla-Pareek SL (2015). Histone chaperones in Arabidopsis and rice: genome-wide identification, phylogeny, architecture and transcriptional regulation. BMC Plant Biol.

[36] Fujimoto S, Sugano SS, Kuwata K, Osakabe K, Matsunaga S (2016). Visualization of specific repetitive genomic sequences with fluorescent TALEs in Arabidopsis thaliana. J. Exp. Bot..

[37] Tirichine L, Andrey P, Biot E, Maurin Y, Gaudin V (2009). 3D fluorescent in situ hybridization using Arabidopsis leaf cryosections and isolated nuclei. Plant Methods.

[38] Hisanaga T (2013). The ATM-dependent DNA damage response acts as an upstream trigger for compensation in the fas1 mutation during Arabidopsis leaf development. Plant Physiol..

[39] Endo M (2006). Increased frequency of homologous recombination and T-DNA integration in Arabidopsis CAF-1 mutants. EMBO J..

[40] Ramirez-Parra E, Gutierrez C (2007). E2F regulates FASCIATA1, a chromatin assembly gene whose loss switches on the endocycle activates gene expression by changing the epigenetic status. Plant Physiol..

[41] Ramirez-Parra E, Gutierrez C (2007). The many faces of chromatin assembly factor 1. Trends Plant Sci.

[42] Benhamed M, Bertrand C, Servet C, Zhou DX (2006). Arabidopsis GCN5, HD1, and TAF1/HAF2 interact to regulate histone acetylation required for light-responsive gene expression. Plant Cell.

[43] Fukaki H, Taniguchi N, Tasaka M (2006). PICKLE is required for SOLITARY-ROOT/IAA14-mediated repression of ARF7 and ARF19 activity during Arabidopsis lateral root initiation. Plant J.

[44] Nelissen H (2010). Plant elongator regulates auxin-related genes during RNA polymerase II transcription elongation. Proc. Natl. Acad. Sci. USA.

[45] Kaya H (2001). FASCIATA genes for chromatin assembly factor-1 in arabidopsis maintain the cellular organization of apical meristems. Cell.

[46] Rosa S (2014). Cell differentiation and development in Arabidopsis are associated with changes in histone dynamics at the single-cell level. Plant Cell.

[47] Mambro (2017). Auxin minimum triggers the developmental switch from cell division to cell differentiation in the Arabidopsis root. Proc. Natl. Acad. Sci. USA.

[48] Mei H (2009). Root development under metal stress in Arabidopsis thaliana requires the H+/cation antiporter CAX4. New Phytol.

[49] Sun P, Tian QY, Chen J, Zhang WH (2010). Aluminium-induced inhibition of root elongation in Arabidopsis is mediated by ethylene and auxin. J. Exp. Bot..

[50] Sakamoto T, Matsunaga S, Fujiwara T (2013). Characterization of the roots of Arabidopsis mutant hypersensitive to boron overload stress. The Proceedings of the International Plant Nutrition Colloquium.

[51] Yuan L, Liu X, Luo M, Yang S, Wu K (2013). Involvement of histone modifications in plant abiotic stress responses. J. Integr. Plant Biol..

[52] Deckert J (2005). Cadmium toxicity in plants: is there any analogy to its carcinogenic effect in mammalian cells?. Biometals.

[53] Rounds MA, Larsen PB (2008). Aluminum-dependent root-growth inhibition in Arabidopsis results from AtATRregulated cell-cycle arrest. Curr. Biol..

[54] Hasegawa J (2014). Increase in invaginated vacuolar membrane structure caused by plant cell expansion by genotoxic stress induced by DNA double-strand breaks. Cytologia.

[55] Kumagai-Sano F, Hayashi T, Sano T, Hasezawa S (2006). Cell cycle synchronization of tobacco BY-2 cells. Nat. Protoc..

[56] Hasegawa J (2016). Three-dimensional imaging of plant organs using a simple and rapid transparency technique. Plant Cell Physiol.

[57] Hirakawa T, Katagiri Y, Ando T, Matsunaga S (2015). DNA double-strand breaks alter the spatial arrangement of homologous loci in plant cells. Sci. Rep..

[58] van Blokland R, ten Lohuis M, Meyer P (1997). Condensation of chromatin in transcriptional regions of an inactivated plant transgene: evidence for an active role of transcription in gene silencing. Mol. Gen. Genet..

[59] Notaguchi M, Higashiyama T, Suzuki T (2015). Identification of mRNAs that move over long distances using an RNA-Seq analysis of Arabidopsis/Nicotiana benthamiana heterografts. Plant Cell Physiol.

[60] Langmead B, Trapnell C, Pop M, Salzberg SL (2009). Ultrafast and memory-efficient alignment of short DNA sequences to the human genome. Genome Biol..

[61] Wilkins O (2016). EGRINs (environmental gene regulatory influence networks) in rice that function in the response to water deficit, high temperature, and agricultural environments. Plant Cell.

[62] Zhang (2008). Model-based Analysis of ChIP-Seq (MACS). Genome Biol..

[63] Shen L, Shao N, Liu X, Nestler E (2014). ngs.plot: Quick mining and visualization of next-generation sequencing data by integrating genomic databases. BMC Genomics.

[64] Du Z, Zhou X, Ling Y, Zhang Z, Su Z (2010). agriGO: a GO analysis toolkit for the agricultural community. Nucleic Acids Res.

