# Supplementary material for: Auxin decreases chromatin accessibility through the TIR1/AFBs auxin signaling pathway in proliferative cells
Source: Sci Rep. 2018 May 17;8:7773. doi: 10.1038/s41598-018-25963-y (PMC5958073; doi:10.1038/s41598-018-25963-y)
Supplement: Supplementary file 1 — Supplementary Information [file 41598_2018_25963_MOESM1_ESM.pdf]

Supplementary Information

Title:
Auxin decreases chromatin accessibility through the TIR1/AFBs auxin signaling pathway in proliferative cells

Authors:
Junko Hasegawa[1#], Takuya Sakamoto[1#], Satoru Fujimoto[1], Tomoe Yamashita[1], Takamasa Suzuki[2], and Sachihiro Matsunaga[1 *]

Affiliations:
[1] Department of Applied Biological Science, Faculty of Science and Technology, Tokyo University of Science, 2641 Yamazaki, Noda, Chiba 278-8510, Japan.
[2] College of Bioscience and Biotechnology, Chubu University, 1200 Matsumoto-cho, Kasugai, Aichi 487-8501 Japan.
#These authors equally contributed to this work.
[*]Corresponding author: Sachihiro Matsunaga, sachi@rs.tus.ac.jp

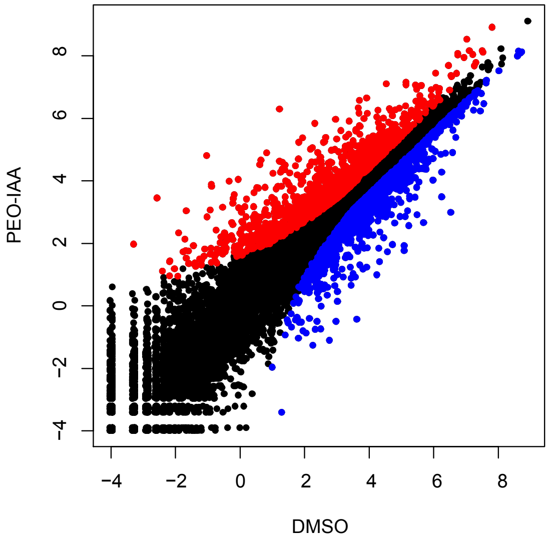

Fig. S1. Effect of PEO-IAA treatment on gene expression in MM2d cells. Scatter plots of log2 (Fragments Per Kilobase of exon per Million mapped fragments) transformed ratios of each transcription unit. Transcripts showing 1.5 ≤ PEO-IAA/DMSO and PEO-IAA/DMSO ≤ 0.67 are shown in red and blue, respectively (FDR < 0.05).

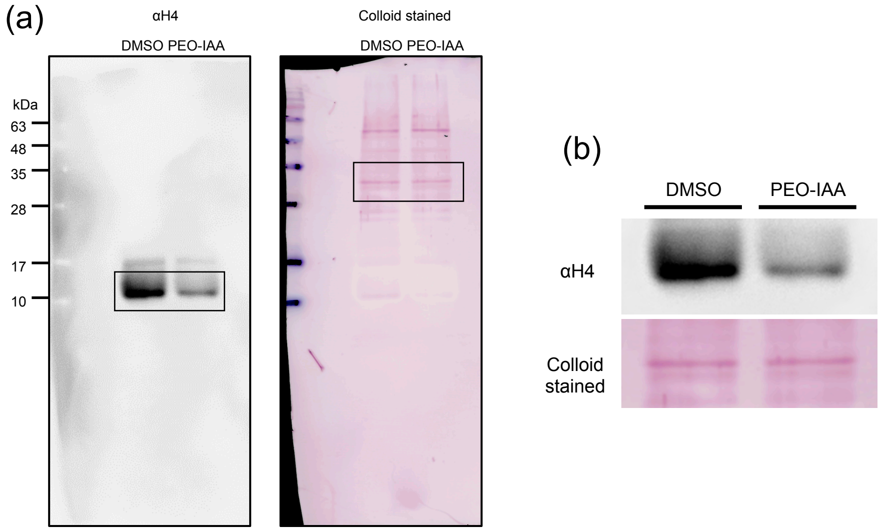

Fig. S2. Effect of PEO-IAA treatment on the amounts of core histone H4. After 2 days PEO-IAA treatment, western blotting analysis with an anti-histone H4 antibody (αH4) was performed**.** (a) Full-length western blot shown in Fig. 3(b). Lanes used are outline with a black rectangle.

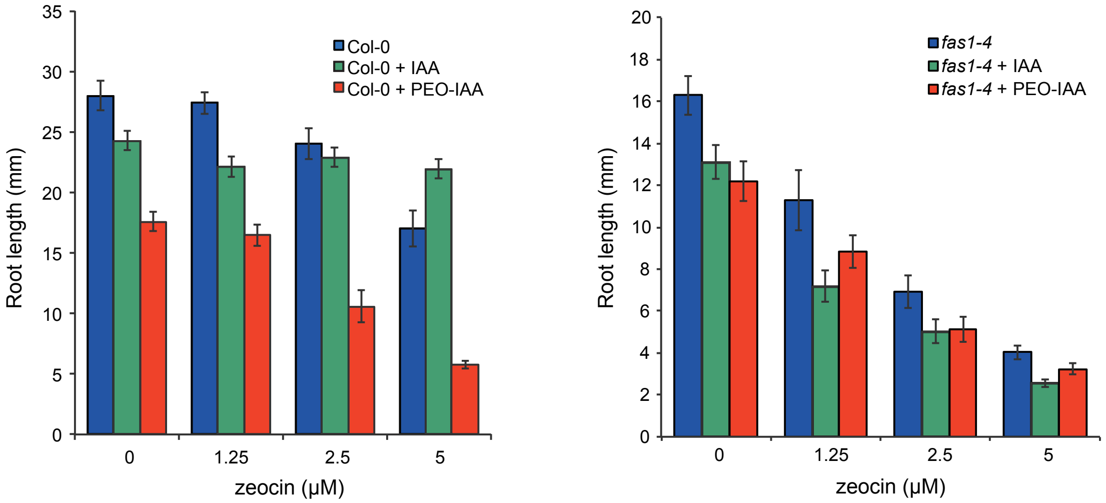

Fig. S3. Effects of 5 nM IAA and 5 μM PEO-IAA on inhibition of root elongation caused by zeocin treatment. Five-day-old seedlings were treated with drugs for 6 days. The primary root length was measured. The sensitivity to zeocin was evaluated by relative value as shown in Fig. 5.

Table S1. Number of sequenced and processed reads

Experiment	Treatment	Sample	Raw reads	Mapped reads
RNA-seq	DMSO	DMSO_1	20466604	17373934
		DMSO_2	22273440	18708662
		DMSO_3	20859028	17507465
	PEO-IAA	PEO_1	19720719	16568763
		PEO_2	12101777	10081073
		PEO_3	21593351	17913588
ATAC-seq	DMSO	DMSO_1	34034741	1285158
		DMSO_2	41214719	1196841
	PEO-IAA	PEO_1	36108392	1354802
		PEO_2	47887156	1159672

Table S2. Number of AuxRE elements in the promoter region (3000 bp upstream) of chromatin-related genes affected by PEO-IAA treatment

Gene		AGI	TGTCNN	TGTCTC	TGTCTD	TGTCVC	GACA
SDG34/SET34/ATXR6		AT5G24330	11	1	1	1	12
CMT2/DMT5		AT4G19020	7	1	2	1	10
CHR20/ATXR		AT1G08600	8	1	3	0	5
NRP2		AT1G18800	5	0	2	1	8
HAC12		AT1G16710	6	0	3	0	6
EBS		AT4G22140	0	0	0	0	2
BRU1/MGO3/TSK		AT3G18730	4	0	1	0	1
SDG18/SET18/SUVR2		AT5G43990	13	1	5	2	8
MET1/DDM2/DMT1		AT5G49160	4	0	1	0	2
CHR25/RAD54		AT3G19210	10	1	3	0	9
CHR16		AT3G54280	15	1	2	1	7
FVE/MSI4		AT2G19520	6	1	1	1	5
CHR17		AT5G18620	10	0	1	3	8
HDT4		AT2G27840	0	0	0	0	3
FAS1		AT1G65470	7	1	0	0	2
CHR1/DDM1		AT5G66750	2	0	0	0	2
AtASF1b		AT5G38110	3	0	0	2	3
UBC3		AT5G62540	3	0	2	0	3
RING1b		AT1G03770	6	3	2	1	3
MET2/DMT2		AT4G14140	9	0	3	0	8
UBC1		AT1G14400	4	0	1	0	1
HFO1	H4	At3g46320	0	0	0	0	3
HFO7	H4	At3g45930	0	0	0	0	3
HTR13	H3.1	At5g10390	5	1	2	0	2
HTA8/H2AZ	H2A.Z.8	At2g38810	8	2	1	1	7
HFO6	H4	At5g59970	14	2	1	1	12
HTB6	H2B.6	At3g53650	15	4	3	1	7
HTB2	H2B.2	At5g22880	1	0	0	1	1
HTA6	H2A.W.6	At5g59870	10	0	3	0	6
HFO2	H4	At5g59690	2	0	0	0	4
HFO3	H4	At2g28740	7	0	1	0	12
HTA11/H2AZ	H2A.Z.11	At3g54560	11	2	3	1	7
HTR2	H3.1	At1g09200	9	0	3	0	2
HTR1	H3.1	At5g65360	4	0	1	0	4
HFO5	H4	At3g53730	2	0	1	0	1
HTB5	H2B.5	At2g37470	10	2	4	0	10
HTA7	H2A.W.7	At5g27670	3	0	0	0	1
HTA2	H2A.2	At4g27230	12	2	5	0	12
HTR3	H3.1	At3g27360	5	3	0	0	5
HTB11	H2B.11	At3g46030	7	0	2	0	11
HTA3	H2A.X.3	At1g54690	4	1	0	1	2
HTA10	H2A.10	At1g51060	11	2	2	1	4
HTB9	H2B.9	At3g45980	6	2	3	0	4
HTA13	H2A.13	At3g20670	11	0	0	3	9
HTR4	H3.3	At4g40030	1	0	1	0	2
